# Supplementary material for: APRF1 Interactome Reveals HSP90 as a New Player in the Complex That Epigenetically Regulates Flowering Time in Arabidopsis thaliana
Source: Int J Mol Sci. 2024 Jan 21;25(2):1313. doi: 10.3390/ijms25021313 (PMC10816710; doi:10.3390/ijms25021313)
Supplement: Supplementary file 1 [file ijms-25-01313-s001.zip › Figure S1.pdf]

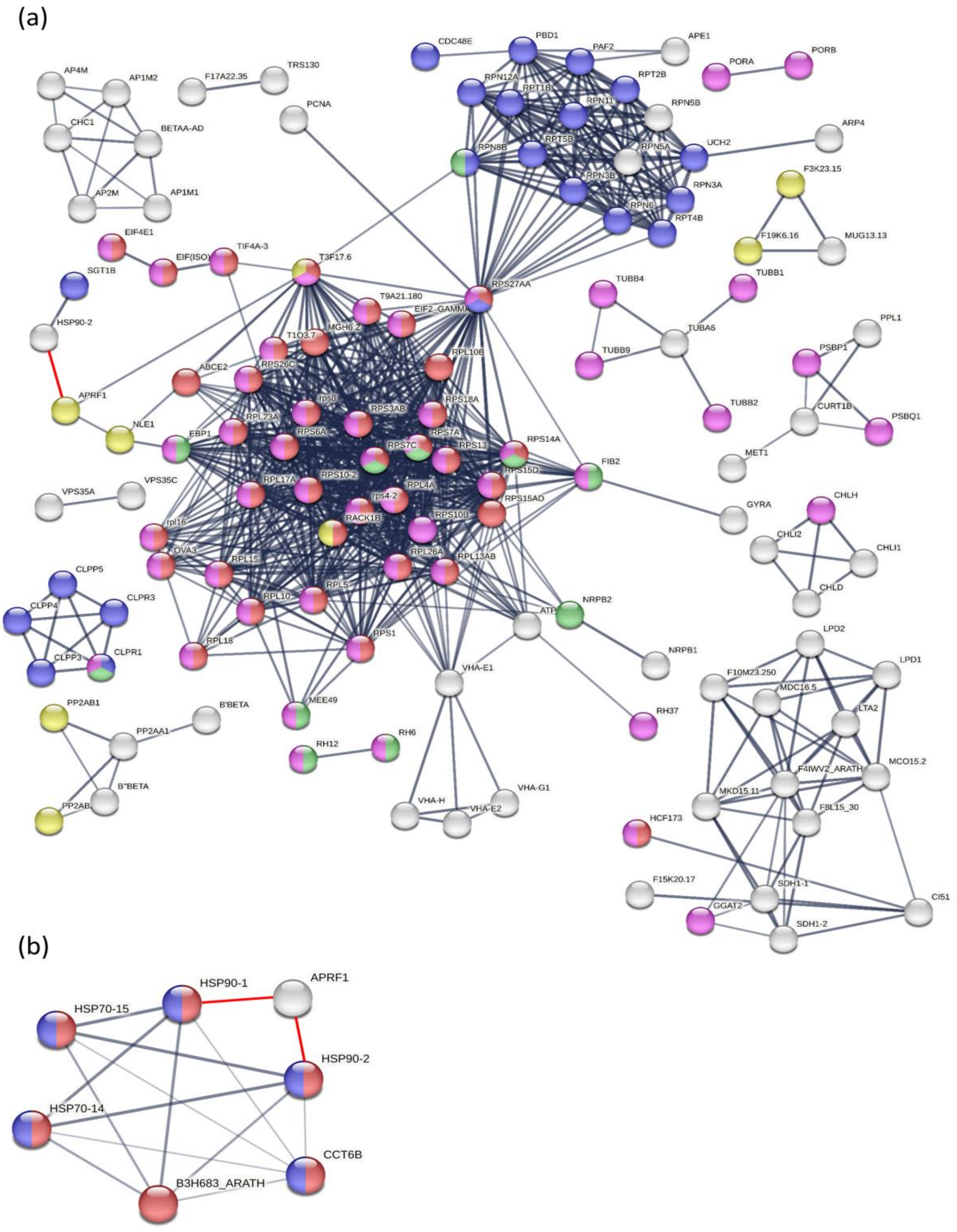

Supplementary Figure S1. Protein-protein interaction analysis via STRING database. (**a**) Network analysis of the proteins belonging to the protein categories listed in Figure 4b and related to the "protein-containing complex", "translation" and "RNA binding", being visualized in a STRING confidence-interaction network (edges). The difference in line thickness represents the strength of the supporting data. Nodes in red indicate proteins related to "translation", in blue those related to "proteolysis", and in green and purple proteins associated with "RNA processing" and "RNA binding", respectively. Proteins containing WD40 repeats are illustrated in yellow. Red-colored edge indicates the novel interaction derived from our study. (**b**) STRING network analysis of proteins belonging, among others, to the "protein folding" and "chaperone" categories. Nodes in red and blue indicate proteins related to "protein folding" and "chaperone" categories, respectively. Edges in red indicate interactions derived from our experiments.
