# Supplementary material for: APRF1 Interactome Reveals HSP90 as a New Player in the Complex That Epigenetically Regulates Flowering Time in Arabidopsis thaliana
Source: Int J Mol Sci. 2024 Jan 21;25(2):1313. doi: 10.3390/ijms25021313 (PMC10816710; doi:10.3390/ijms25021313)
Supplement: Supplementary file 1 [file ijms-25-01313-s001.zip › Table S2.pdf]

Supplementary Table S2: List of primers used in this study.

Primer name	Sequence
WUS-F	TGCAAGCTCAGGTACTGAATG
WUS-R	ACGTCGATGTTCCAGATAAGC
STM-F	CATAACGAAATCGTTGCAGGA
STM-R	ATCGACTTCTTCCTCGGATGA
CLV1-F	GCTTGCTAAGTTCTTAGTTGATGG
CLV1-R	GCATCCGATGGCTGAGTTAT
GAPDH-F	TGTCAACGAGCACGAATACAAG
GAPDH-R	TCACAAACTTGTCGCTCAATGC
APRF1-Xba-SpyNE	ATATCTAGATGTCGTTGACGGAGCTAGAC
APRF1-Xho-SpyNE	ATACTCGAGCTGTTGTTGATCGGTAGGAGGA
APRF1-FLAG-F	TAATCTAGATGGTGGAATTTACAGAGAGAG
APRF1-FLAG-R	TATGAGCTCTAATCCTTGTAGTCCTTGTCATC GTCATCCTTGTAGTCTTTCCGAATGGCATAA GCTGGC
